# Induction of labor in twin pregnancy in patients with a previous cesarean delivery

**DOI:** 10.1186/s12884-023-05868-z

**Published:** 2023-07-26

**Authors:** Aharon Dick, Naama Lessans, Gabriel Ginzburg, Einat Gutman-Ido, Gilad Karavani, Hila Hochler, Yael Suissa-Cohen, Joshua I Rosenbloom

**Affiliations:** 1 Department of Obstetrics and Gynecology, Hadassah Medical Organization, Faculty of Medicine, Hebrew University of Jerusalem, Jerusalem, Israel; 2 Hebrew University Medical School, Jerusalem, Israel

**Keywords:** Induction of labor, Trial of labor after cesarean delivery, Twin gestation

## Abstract

**Background:**

Trial of labor after cesarean delivery (TOLAC) in twin gestations has been associated with decreased rates of successful vaginal delivery compared to singleton pregnancies, with mixed results regarding maternal and neonatal morbidity. However, induction of labor (IOL) in this unique population has not yet been fully evaluated.

**Objective:**

To assess success rates and maternal and neonatal outcomes in women with a twin gestation and a previous cesarean delivery undergoing IOL.

**Methods:**

A retrospective cohort study including women with a twin gestation and one previous cesarean delivery undergoing a trial of labor between the years 2009–2020. Patients requiring IOL were compared to those with a spontaneous onset of labor.

**Results:**

There were 53 patients who met the inclusion criteria: 31 had a spontaneous onset of labor (58%) and 22 required an IOL. Baseline characteristics were comparable between the groups apart from a history of labor arrest which was more common in the IOL group (40.9% vs. 9.6%, P = 0.006). A successful vaginal delivery occurred in all (100%) women with a spontaneous labor compared to 81% in the IOL group (p = 0.02). Secondary outcomes were comparable. A history of no previous vaginal delivery, maternal obesity, and IOL were associated with TOLAC failure.

**Conclusions:**

IOL after cesarean delivery in twin gestation is associated with an increased risk of TOLAC failure compared to spontaneous onset of labor. However, no adverse neonatal or maternal outcomes were found. IOL in this high-risk population is feasible but patients should be counseled about the lower rate of success.

## Background

The rising rates of cesarean delivery globally (CD) have brought renewed efforts to promote trial of labor after cesarean delivery (TOLAC) [1]. Due to the known risks associated with CD, including neonatal and maternal morbidities [2, 3], as well as the increased risk for future obstetrical complications, efforts are being made to reduce the rate of repeat cesarean deliveries and to characterize the unique population that is most suitable for TOLAC.

Induction of labor (IOL) in women with a previous cesarean delivery is associated with a higher incidence of failed TOLAC and uterine rupture compared to spontaneous labor [1]. Additionally, the risk of other maternal morbidities such us the need for blood transfusion, thromboembolism, and hysterectomy is increased [4, 5].

Although there has been a decreased incidence in recent years [6], twin gestation still comprises roughly 3% of all pregnancies. The majority of women carrying a twin gestation with a history of a previous CD will choose an elective repeat CD [7]; however, TOLAC is not contraindicated.

Several studies have evaluated success rates and safety of a TOLAC in twin gestation; there is no unanimity of opinion regarding the success of rates of vaginal delivery. A number of studies have indicated similar success rates compared to TOLAC in singleton pregnancies with an increased risk of complications [8, 9], while others found lower success rates [10, 11].

Oxytocin administration in singleton TOLAC has been associated with an increased risk of uterine rupture [12] and emergent CD. Nevertheless, a recent cohort study examining factors associated with failed TOLAC in twin gestation found that the use of oxytocin was associated with increased rates of TOLAC success [13].

However, not all patients who are interested in TOLAC with a twin pregnancy will enter into spontaneous labor. To date, data regarding success rates of IOL in this unique population are scarce.

Therefore, the purpose of this study was to evaluate the safety and effectiveness of IOL in twin gestation in women with a previous CD, compared to women with a spontaneous onset of labor attempting TOLAC.

## Methods

### Population

This is a retrospective cohort study at two campuses of a large academic medical center of women with a history of one previous CD and a current twin gestation undergoing a trial of labor between 2009 and 2020. Women who had an elective repeat CD, or who presented in active labor or with ruptured membranes but who did not wish to proceed with a trial of labor were excluded from the study.

Other exclusion criteria included women with a contraindication for vaginal delivery such as non-vertex presentation of the presenting twin, a history of previous classical CD or other uterine surgery, or a history of more than one prior CD.

The IOL group included women receiving oxytocin, artificial rupture of membranes (AROM) or a combination of the two before entering active labor (i.e. dilatation of less than 6 centimeters). Criteria for IOL due to gestational age in our hospital are 37–38 weeks in dichorionic pregnancies and 36–37 weeks in monochorionic-diamniotic pregnancies [14].

Women who presented in active labor and underwent AROM for technical reasons (i.e. connecting an internal monitor to the leading fetus) or during the second stage of labor, were not considered as undergoing IOL and were included in the spontaneous onset group.

Our protocol for oxytocin in women undergoing TOLAC (both singleton and twins) recommends an initial dose of 1 miU/min. At 30-min intervals, the dose is gradually increased by increments of 1 miU/min until a maximum of 20miU/min or when a desired contraction pattern is established.

Maternal and neonatal data were retrieved using a computerized database, continuously updated and validated for admission, labor, and postpartum course. Data collected included maternal age, body mass index (BMI), gravidity, parity, history of previous vaginal delivery or VBAC, maximum birthweight in any prior vaginal delivery, indication for previous CD, birthweight at previous CD, and time interval between the prior delivery and the current delivery.

Data regarding current labor included gestational age (determined by early ultrasound), chorionicity, presence of gestational hypertension or preeclampsia, gestational diabetes, indication for IOL, epidural analgesia and neonatal birthweight.

### Outcomes

Our primary outcome was the rate of successful vaginal delivery of twin A (the leading twin). Secondary outcomes were other delivery outcomes including vacuum assisted delivery, postpartum hemorrhage (defined as estimated blood loss > 500 mL), chorioamnionitis and uterine rupture. Neonatal outcomes examined were low umbilical PH (< 7.1) and low Apgar score (< 7).

### Statistical analysis

Continuous variables with normal distribution were compared using Student’s t-test. The continuous variables without a normal distribution were compared using the Mann–Whitney U test. Categorical variables were compared using Pearson’s chi-square or Fisher’s exact test as appropriate. We also conducted a secondary analysis aiming to identify factors associated with a successful TOLAC. A p value of < 0.05 was considered statistically significant. Statistical analysis was performed using IBM SPSS Statistics for Windows, version 24.0 (Armonk, NY, USA). The local Institutional Review Board approved the study protocol.

## Results

During the study period, 352 women with a twin gestation and one previous CD delivered in our center, yet only 53 (15%) attempted a trial of labor. Of these, 31 (58%) women had a spontaneous onset of labor and 22 (42%) required an IOL. Half (n = 11) of the women in the study group were induced with oxytocin, while the rest were induced with AROM alone.

Baseline characteristics are presented in Table 1. The groups were comparable regarding maternal age, BMI, gravidity, parity and history of previous VBAC. The majority of women in both groups had a previous VBAC. The most common indication for previous CD was non vertex presentation (26%, n = 14), followed by non-reassuring fetal heart rate (25%, n = 13) and protracted labor (arrest of dilatation or descent, 23% n = 12). A history of labor arrest was more common in the IOL group (40.9% vs. 9.6%, p = 0.006).

**Table 1:** Baseline Characteristics

Variable	Spontaneous labor (n = 31)	IOL (n = 22)	P
Age (years)	32 (27–36)	32 (28–36)	0.75
BMI (kg/m^2^)	31.08 (6.20)	28.68 (4.26)	0.27
Arrest of labor as indication for previous CD	3 (9.6%)	9 (40.9%)	0.006
Gravidity	4 (3–6)	4 (3–6)	0.97
Parity	3 (2–5)	2 (1–4)	0.98
Primi-parity	7 (22.5%)	9 (40.9%)	0.15
Any past VBAC	20 (64.5%)	12 (54.5%)	0.55
No. of previous VBAC	1 (0–3)	1 (0-2.5)	0.97
Birthweight in previous CD (gr)	2833.5 (640.8)	2892.0 (702.7)	0.61
Maximal previous vaginal delivery birthweight (gr)	3130.9 (386.2)	3432.7 (422.1)	0.11

Numbers are n (%) or median (interquartile range) or mean (standard deviation)

CD- cesarean delivery; VBAC- vaginal birth after Cesarean delivery

Current pregnancy and labor characteristics are presented in Table 2. Gestational age, chorionicity, interval from previous CD, history of hypertension or diabetes during pregnancy and use of epidural analgesia during labor were comparable between the groups. The most common indication for IOL was gestational age (68%, n = 15).

**Table 2:** Pregnancy Characteristics

Variable	Spontaneous labor (n = 31)	IOL (n = 22)	P
Gestational age, weeks	37.4 (35.2–38.4)	38.5 (37.3–39.4)	0.10
Dichorionic pregnancy	23 (74.1%)	20 (90.9%)	0.10
Hypertensive disorder of pregnancy	0 (0%)	2 (9%)	0.16
Gestational diabetes	2 (6.4%)	0 (0%)	0.50
Epidural analgesia	20 (64.5%)	16 (72.7%)	0.52
Interval between CD and TOLAC (years)	5 (2–6)	5 (3-7.75)	0.47
Birthweight twin A (gr)	2481.2 (410.7)	2720.2 (295.9)	0.21
Birthweight twin B (gr)	2586.2 (443.9)	2736.1 (389.7)	0.26

Numbers are n (%) or median (interquartile range) or mean (standard deviation)

CD- cesarean delivery; TOLAC- trial of labor after cesarean delivery

The delivery outcomes are presented in Table 3. A successful vaginal delivery (including instrumental delivery) occurred in all (100%) women with a spontaneous labor compared to 81% in the IOL group (p = 0.02).

**Table 3:** Outcomes

Variable	Spontaneous labor (n = 31)	IOL (n = 22)	P
Successful VBAC	31 (100%)	18 (81.8%)	0.02
VAD TWIN A	3 (9.6%)	4 (18.1%)	0.43
TBE TWIN B	11 (35.4%)	10 (45.4%)	0.46
PPH	4 (12.9%)	1 (4.5%)	0.38
Twin A APGAR < 7	0 (0%)	1 (4.5%)	0.415
Twin B APGAR < 7	0 (0%)	1 (4.5%)	0.415
Twin A umbilical artery pH < 7.1	0 (0%)	1 (4.5%)	0.415
Twin B umbilical artery pH < 7.1	0 (0%)	0 (0%)	
Uterine rupture	0 (0%)	0 (0%)	
Chorioamnionitis	0 (0%)	0 (0%)	

Numbers are n (%)

VBAC- vaginal birth after cesarean delivery; VAD- vacuum assisted delivery; TBE- total breech extraction; PPH- postpartum hemorrhage

Secondary outcomes including postpartum hemorrhage or vacuum assisted delivery did not differ between the groups. No cases of uterine rupture or chorioamnionitis occurred in either of the groups. No differences were found regarding neonatal adverse outcomes such as low Apgar score or low umbilical pH.

In a secondary analysis aiming to identify factors associated with a successful TOLAC, IOL, absence of prior vaginal delivery, maternal obesity and high birthweight were all more common in the failed TOLAC group (Table 4**).** Moreover, arrest of labor as indication for previous CD was more common in the failed TOLAC group, yet this did not reach statistical significance.

**Table 4:** Factors Associated with Successful TOLAC

	Successful TOLAC (n = 49)	Failed TOLAC (n = 4)	P
Age (years)	32 (27.5–36)	33 (28.5–36)	0.80
BMI (kg/m^2^)	28.88 (5.24)	33.07 (2.24)	0.03
Arrest indication for previous CD	9 (18.3%)	3 (75%)	0.05
Gravidity	4 (3–6)	2.5 (2-3.75)	0.19
Parity	3 (1–4)	1 (1-1.75)	0.34
No prior vaginal delivery	9 (18.3%)	3 (75%)	0.009
Any past VBAC	31 (63.2%)	1 (25%)	0.12
No. of previous VBAC	1 (0–3)	0 (0-1.5)	0.94
Birthweight in previous CD (gr)	2778.14 (638.34)	3595.0 (318.48)	0.007
Gestational age, weeks	38 (36.5–36.8 )	38.28 (35.96–39.53)	0.80
Dichorionic pregnancy	41 (83.6%)	2 (50%)	0.37
Hypertensive disorder of pregnancy	1 (2.04%)	1 (50%)	0.14
Gestational diabetes	2 (4.08%)	0 (0%)	1.00
Epidural analgesia	33 (67.3%)	3 (75%)	0.75
Interval between CD and TOLAC (years)	5 (3–6)	5 (3–5)	0.42
IOL	18 (36.7%)	4 (100%)	0.02

Numbers are n (%) or median (interquartile range) or mean (standard deviation)

CD- cesarean delivery; VBAC- vaginal birth after cesarean delivery; TOLAC- trial of labor after cesarean delivery; IOL- induction of labor

## Discussion

In this study we report the outcomes following IOL in twin gestation in women with a previous CD. We found that compared to women with spontaneous onset of labor, IOL was associated with decreased rates of a successful TOLAC. The incidence of maternal or neonatal adverse outcomes, such as PPH, instrumental delivery, low five-minute Apgar score and low umbilical artery pH did not differ between the groups. Spontaneous onset of labor, prior vaginal delivery, lower maternal BMI and lower birthweight were all associated with a successful TOLAC.

Although there are a plethora of studies regarding the safety of IOL for TOLAC in singleton gestation, and a number of studies regarding the safety of TOLAC in twin gestation, to our knowledge there are few if any studies specifically addressing the combination of both IOL and twin gestation on TOLAC success and safety. Regarding IOL in singleton TOLAC, there is a known increased risk of uterine rupture and failed TOLAC with the use of oxytocin [15, 16]. As for twin TOLAC, a recently published cohort study found that women with a twin gestation undergoing TOLAC were less likely to have a successful VBAC when compared to singleton TOLAC. Additionally, higher rates of uterine rupture and other maternal complications were found in that study [11]. On the other hand, a meta-analysis published in 2019 that included over 2400 women with a planned TOLAC found that no increased risk of uterine rupture or failed TOLAC among women with twin gestation compared to those with a singleton pregnancy [17]. These mixed findings and clinician uncertainty regarding the safety of TOLAC in twin gestation may explain the low rates of TOLAC in these patients: studies have reported that only 12–30% of women in this situation pursue a vaginal delivery [10, 11].

Evidence of IOL success rates in twin gestation and a previous CD are scarce. Our results provide a preliminary insight to this topic. Indeed, we found that induction of labor in this population was highly successful and no adverse maternal or neonatal outcomes were found. Unsurprisingly, success rates of TOLAC in spontaneous onset of labor were higher. Larger scale studies are required to reinforce our conclusion. Moreover, nearly 70% of our cohort were women with a previous vaginal delivery, which is a known factor for TOLAC success. Therefore the relevance of our findings to women with no prior vaginal delivery is questionable.

The main strength of our study is its originality: to the best of our knowledge we are the first to specifically evaluate IOL success rate in women with a twin gestation and a previous CD. Furthermore, our study relied on a robust labor and delivery database with detailed clinical and demographic data.

Apart from its retrospective design, our study has several limitations: first, we were underpowered to assess incidence of some adverse outcomes, mainly uterine rupture, thus limiting our ability to assess safety. Second, we had a relatively small cohort, limiting generalizability of our results. Third, indication for previous CD was clearly different between the groups, perhaps cofounding our results.

Finally, the true choice available to the clinician in this scenario is not the choice between IOL and spontaneous labor; it is between IOL, repeat cesarean, or expectant management [18]. However, we chose to evaluate outcomes in spontaneous labor compared to IOL because we assume that few clinicians would offer IOL to TOLAC patients carrying twins without a clear-cut indication. Therefore, for these patients a choice of IOL vs. expectant management is not the usual clinical choice- it is either IOL or repeat CD.

## Conclusion

IOL in twin gestation in women with a previous CD is associated with decreased rates of successful vaginal delivery compared to spontaneous onset of labor. However, overall IOL in these patients is generally successful. No cases of uterine rupture or adverse neonatal outcomes were found. Larger scale studies are needed to assess safety of this intervention.

## Data Availability

The de-identified datasets used and/or analyzed during the current study are available from the corresponding author on reasonable request.

## Abbreviations

AROM: Artificial rupture of membranes
CD: Cesarean delivery
IOL: Induction of labor
PPH: Postpartum hemorrhage
TOLAC: Trial of labor after cesarean delivery
